# Pseudotumor inflammatory of the spleen: A rare entity with diagnostic and therapeutic challenges: A case report

**DOI:** 10.1016/j.ijscr.2023.109215

**Published:** 2023-12-31

**Authors:** Hamza Ouzzaouit, Boubker Idrissi Kaitouni, Fouad Zouaidia, Sekkat Hamza, Abdelmalek Hrora, Mohammed Raiss

**Affiliations:** a Digestive Surgical Department C, Ibn Sina University Hospital, Rabat, Morocco; b Faculty of Medicine and Pharmacy, Mohammed V University in Rabat, Morocco; c Anatomopathology department, Ibn Sina University Hospital, Rabat, Morocco

**Keywords:** Spleen, Inflammatory pseudotumor, Rare entity, Surgery

## Abstract

**Introduction and importance:**

Inflammatory pseudotumor (IPT) of the spleen is a rare entity that can be difficult distinguishing it from malignancies, both in clinical presentation and radiological imaging.

**Case presentation:**

We present the case of a 43-year-old female presented with 15-cm left hypochondrial mass, Initial imaging studies raised concerns of malignancy, leading to a splenectomy. However, the final pathological examination determined that the patient had IPT of the spleen with focal expression of Smooth Muscle Antibody (SMA).

**Clinical discussion:**

This case highlights the importance of considering IPT as a potential diagnosis of splenic masses that was difficult to diagnose before surgery.

**Conclusion:**

The uniqueness of the case under consideration lies in the rarity and the atypical localization of IPT of the spleen.

## Highlights

• Inflammatory pseudotumor (IPT)of the spleen is a very rare entity.
• Due to their strong resemblance to malignancies on imaging, IPT of the spleen diagnosis is often challenging.
• Splenectomy is often carried out and the final diagnosis is determined by pathological examination.

## 1 Introduction

Inflammatory pseudotumors are benign conditions with unknown etiology [1,2].They manifest as localized lesions in the spleen, characterized by the presence of fibroblastic growths and infiltration of various types of inflammatory cells, all of which are reactive in nature. All organs can be affected but they tend to occur more in the orbits and the respiratory system.

Splenic localization is extremely rare and only a few cases have been reported in the literature [3]. Despite advancements in imaging technologies, preoperative diagnosis of inflammatory pseudotumor of the spleen (IPT) remains a challenging task and is seldom achieved [4].

By using the 2023 Scare model [24], we report a case of a patient presenting an IPT of the spleen treated by splenectomy, with a literature review.

### 1.1 Presentation of case

A 43-year-old woman without any medical history, reported an upper left quadrant abdominal pain over the last year. Physical examination revealed a 15 cm solid mass, fixed to the left hypochondrium, her blood test was normal, except for a microcytic anemia with Hemoglobin level of 7 g/dl.

**Fig. 1:**
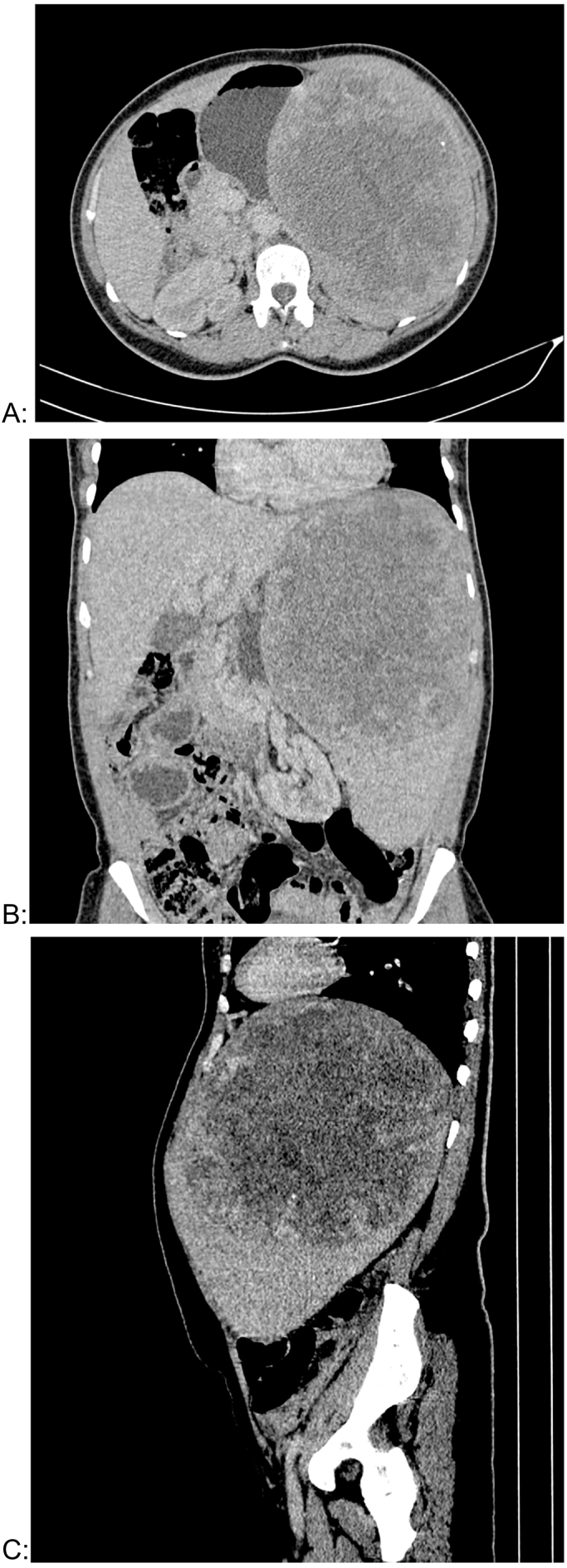
A Computed tomography imaging showing the spleen mass.

Ultrasonography and injected abdominal CT-scan showed a voluminous splenic mass of superior polar origin, roughly rounded, with well-defined boundaries, with clean walls presenting an irregular and nodular way enhancement, and the central area within the mass appeared necrotic and did not enhance with contrast. The mass pushes the left liver and stomach without signs of invasion and the splenic hilum was displaced downward with an intact splenic pedicle, and the left kidney was displaced and positioned medially (Fig. 1).

With such findings splenectomy was recommended. Due to the size of the mass, a laparoscopic approach was impossible, leading to the adoption of a median supra umbilical incision, while surgical exploration found a large splenic mass, in contact with the splenic hilum, with dilatation of the splenic vein.

**Fig. 2:**
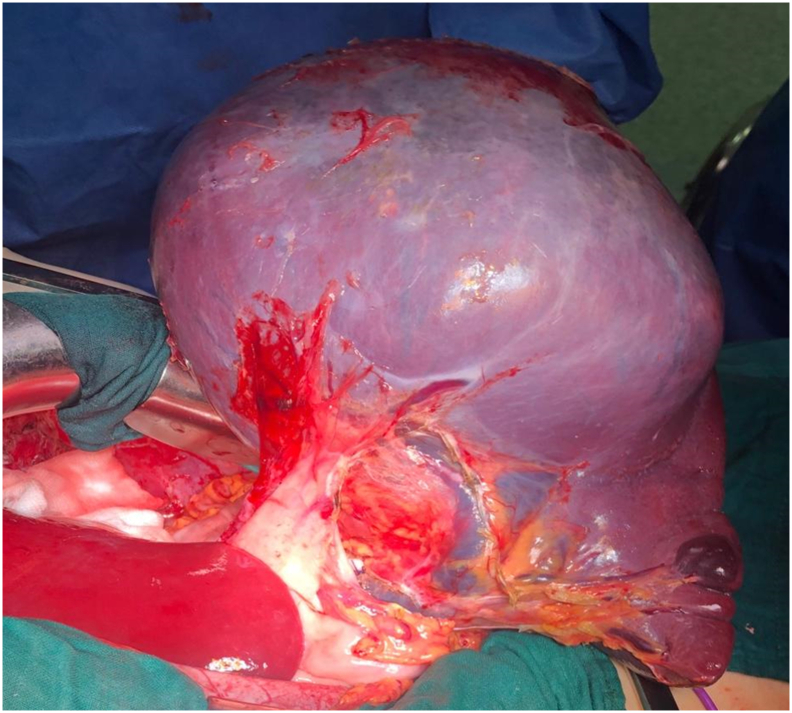
Per operative picture after removing the spleen from abdominal cavity.

We first started by dissecting and ligating the splenic artery while leaving the spleen in place, then the spleen was mobilized extra abdominally after releasing all its peritoneal attachments. Finally, the splenic vein was ligated and sectioned and the specimen was sent to a pathologist (Fig. 2).

**Fig. 3:**
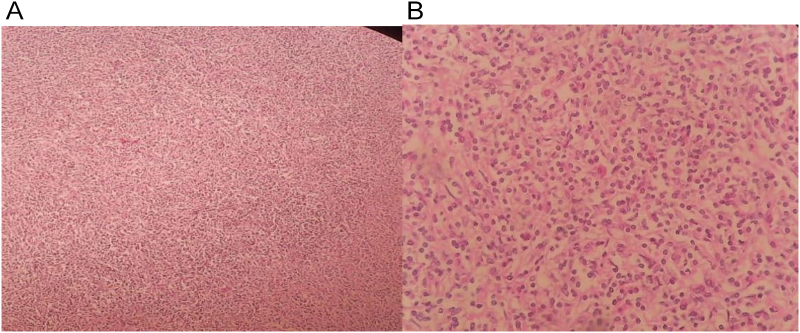
Histological imaging showing splenic parenchyma extensively altered by hyaline fibrosis, with necrotic foci. A (Left): Cellular nodules within abondant fibrohyaline stroma (HE ×100). B (Right):Plasma cells dispersed in hyaline fibrosis (HE ×400).

Anatomopathologist received a splenectomy weighing 2 kg 800 g and measuring 30 × 18 × 10 cm. On section, there was a heterogeneous yellowish-white lesion measuring 21 × 12 × 7 cm, and histological analysis revealed that the splenic parenchyma had been replaced by hyalinized fibrosis with areas of necrosis and lymphoid aggregates. Immunohistochemical study showed an intense labeling of spindle cells with the anti-smooth muscle actin (SMA) antibody, while they were negative for the Follicular dendritic cell (FDC) markers CD 23 and CD 21 as well as for the anti-ana- plastic-kinase lymphoma (ALK) and anti-latent-membrane protein (LMP1) antibodies. The diagnosis of IPT has been retained (Fig. 3).

The patient's postoperative recovery was uneventful, she received her vaccines against encapsulated germs and she was discharged on the 5th day after surgery. A 10-months follow-up found a patient in good health without showing any kind of complications.

## 2 Discussion

Inflammatory pseudotumors are benign conditions that can manifest in different locations including orbit, liver, respiratory and digestive tracts [1,2].

IPT of the spleen is a rare entity characterized by the presence of inflammatory tissue within the spleen, described the first time by Cotelingam and Jaffe in 1984, and until 2016, only 116 cases had been reported (Table 1) [1,3]. This condition affects mostly women with a mean age of 47.2 years, although rare cases have been reported in children [4,5].

**Table 1:** Clinic, radiology and pathology of the reported cases.

Authors	Number of cases	Age	Sex	Symptoms	Laboratory	Radiological findings	Preoperative Biopsy	Histology	Follow up
Yan et al. [1]	2	60–77	M-F	Abdominal discomfort.Weight loss.	Elevated immunoglobulin G and B-2 microglobulin .	CT: Low density hypovascular mass. Central No necrosis.	No	Necrotic areas. Mix of inflammatory cellular elements, predominantly plasma cells and lymphocytes .	3–10 months.
Martinez Celeda, et al. [2]	1	37	F	Abdominal discomfort. Weight loss.General syndrome.Asymptomatic.	Normal	CT: Focal splenic injury: hypodense, homogeneous, with peripheral uptake.	No	Plasma cells, lymphoid elements and occasional eosinophils	Asymptomatic
Ma et al. [4]	1	77	M-F	Asymptomatic.	Normal	CT-MRI: Splenic mass with diffuse heterogeneous enhancement .	No	Necrotic focus in the center, with admixture of inflammatory cellular elements, predominantly plasma cells and lymphocytes with hyalinization, fibrosis, lymph follicles and multinucleated giant cells.	4 months . Asymptomatic .
Rosenbaum, et al. [11]	1	33–60	M-F	Abdominal pain. Weight loss. Night sweats.	NA	CT: Splenic mass.	No	Necrotic foci. Mixed inflammatory infiltrate with abundant mature plasma cells and a proliferation of spindled cells. Abundant EBV-infected cells that included the proliferating oval and spindle cells.	17–9 months . Asymptomatic.
Noguchi et al. [16]	1	72	F	Nausea.	Normal	CT: Partially calcified, low-density, hypo-vascular, well-defined, smooth mass. MRI: Splenic mass with low to isointensity on T1 and high intensity with surrounding low intensity on T2. Low intensity in the center of the lesion. Angiography: Hypovascular area in the arterial phase.	No	Focus of necrosis in the center, with admixture of inflammatory cellular elements, predominantly plasma cells and lymphocytes with hyalinization, fibrosis, lymph follicles and multinuclear giant cells.	24 months . Asymptomatic .
Matsubayashi et al. [17]	1	61	F	Nasal bleeding.	Pancytopenia.Hepatitis C virus antigen-antibody +	CT: Splenomegaly. Tumor with nodule-in-nodule pattern, with low density outer nodule and high density inner nodule. High contrast enhancement in the early phase in the inner nodule. MRI: Low intensity inner nodule on T1. On T2 low intensity outer nodule with a highly intense inner nodule. Angiography: Cotton-wool appearance.	No	Granulomatous component, with large amount of giant cells, plasma cells, lymphocytes and fibroblast. Inner mass with histology of cavernous hemangioma.	NA
Hamdi et al. [18]	1	31	F	Abdominal pain.	Normal	US: Hypoechoic, heterogeneous splenic lesion. MRI: Isointense on T1.Heterogenous mass with multiple hypointense partitions in radial layout on T2	No	Admixture of inflammatory cellular elements represented for plasma cells, eosinophils and histiocytes.	NA
Yano et al. [19]	2	48	F-M	Abdominal pain . Diarrhea. Asymptomatic.	Leukocytosis. Elevated CRP.	CT: Low density splenic mass. MRI: Low to isointensity on T1. Irregular intensity on T2	No	Infiltration of plasma cells, lymphocytes and histiocytes.	NA
Hrora et al. [20]	1	56	F	Abdominal pain .	NA	CT: Splenic mass with tissular density expense of the lower pole of the spleen, with tissue density, taking up contrast with a central hypodense area. The rest of the splenic parenchyma was normal in appearance as well as the splenic hilum. The tumor was displacing the left kidney downwards without invading it. There was no detectable adenopathy.	No	Mixed Inflammatory infiltrat, Monocytes, Lymphocytes snd Histiocytes . Vascular lesion: Thrombosis.	NA
Bhatt et al. [12]	1	31	M-F	NA	NA	CT: Hypodense splenic lesion with early enhancement . MRI: Isointense on T1 . Low intensity on T2 .	No	Mixed inflammatory infiltrate of lymphocytes, plasma cells and occasional eosinophils.	NA
Moriyama et al. [21]	67	51.2 + −17.5	M:29 F:38	Abdominal pain: 21 Incidental: 38	Anemia or Leukocytosis: 25	US (*n* = 37): Hypo: 30, High: 3, Others:4 CT (*n* = 53): Iso or low: 36, High: 2, Others: 15, Enhanced: 15, Calcification: 5 MRI (*n* = 24): Low: 17, High: 4, Others: 3, Enhanced: 8 Angiography (*n* = 21): Avascular: 17, Hypervascular: 4	No	NA	NA

CT, computed tomography; MRI, magnetic resonance imaging, US: Ultrasonography

The precise etiology of this condition remains unknown, several hypotheses have been proposed, including infection, ischemia, trauma, and autoimmune factors [3].

The inflammatory pseudotumor of the spleen was initially described as a proliferation of fibroblastic spindle cells associated with inflammatory cells [1]. Arber et al. demonstrated that spindle cells can be either myofibroblasts (actin-positive smooth muscle) or FDC that may be infected by Epstein-Barr Virus (EBV) [6].

Further studies have confirmed the heterogeneity of lesions categorized under the term “inflammatory pseudotumor”, encompassing true inflammatory pseudotumors, Inflammatory myofibroblastic tumors (IMT) and inflammatory pseudotumor-like tumors [22].

The presence of necrosis in these lesions suggests an infectious origin although bacteriological examinations and cultures have failed to identify any germ [6]. Various characteristics such as necrotic, hemorrhagic, fibrous or cystic areas have been described [7].

IPT are often asymptomatic, and approximately half of the reported cases were accidentally discovered or during examinations for another disease, such as Hodgkin's disease, idiopathic thrombocytopenic purpura, or hypercalcemia [6,[8], [9], [10]].

Although, they can occasionally manifest with non-specific clinical symptoms, like abdominal pain, fever or weight loss. Clinical examination reveals splenomegaly in 80 % of symptomatic patients as seen in the case presented [1,4,11].

Laboratory findings are usually normal, although hypercalcemia, monoclonal peaks in the proteinogram and polyclonal hypergammaglobulinemia have been reported [1,11].

Due to their strong resemblance to malignancies on imaging, IPT diagnosis is often challenging and conclusive only after splenectomy [8]. Franquet et al. reported that the presence of a central stellate area, corresponding to a fibrous plaque after contrast administration strongly suggests an IPT [23].

While CT scans usually reveal a solitary hypodense nodule, which is hypovascular, and sometimes with a central scar [12], MRI may reveal a well-defined mass, isointense on T1-weighted images, and with varying signal intensity on T2, with respect to the surrounding normal spleen [1,4,14].

Preoperative biopsy of the spleen is not recommended due to its low specificity and the risk of bleeding or tumor dissemination in case of malignancy [1], therefore, splenectomy with subsequent evaluation of the surgical specimen is recommended [2,5].

The treatment of inflammatory pseudotumors of the spleen remains surgical, consisting on a total splenectomy, which is the most common approach in the majority of cases reported in the literature. [8] Partial splenectomy, although rarely performed, may be indicated if the splenic tumor is isolated, located far from the hilum, respecting the capsule, and does not invade neighboring organs or nearby lymph nodes [8,15].

Anatomopathological examination allows ruling out primary splenic lymphomas and sarcomas, which are exceedingly rare. Upon histological examination, the inflammatory pseudotumor exhibits well defined boundaries and develops in the red pulp, encompassing islands of white pulp [18].

Cotelingam describes three histological aspects: hypervascularized myxoid foci, compact areas of spindle cells simulating the cellular regions seen in fasciitis or fibromatosis, and large areas of collagenized hypocellular fibrosis [3].

The cellular composition can display significant heterogeneity and share similarities to granulation tissue, notably, normal lymphocytes and plasma cells are consistently present, although their mixture and quantity may vary, and furthermore neutrophilic and eosinophilic leukocytes can also be observed. Despite attempts to classify these lesions, the terminology used to describe this entity is still confusing, and some authors have categorized these lesions into three histopathological subtypes: xanthogranuloma type, plasma cell granuloma type, and sclerosing pseudotumor [6,15,16].

Immunohistochemical studies have shown strong immunoreactivity of spindle cells to vimentin and SMA, thus confirming their myofibroblastic nature [22].

The prognosis after splenectomy is generally favorable, and local invasion, recurrence, or metastasis have not been reported. However, clinical follow-up is recommended, as there have been cases of patients with inflammatory pseudotumors of the liver who have experienced disease related complications [1,2,16].

**In conclusion**, our case report highlights the importance of considering pseudotumors in the differential diagnosis of splenic masses . Although rare, these lesions can mimic malignant tumors, and accurate diagnosis is crucial for appropriate management . Immunohistochemistry can be a useful tool in the diagnosis of pseudotumors and further studies are needed to better understand the pathogenesis of these lesions .

## Consent of publication

Written informed consent was obtained from the patient for publication of this case report and accompanying images. A copy of the written consent is available for review by the Editor-in-Chief of this journal on request.

## Ethical approval

Ethical approval is exempt/waived at our institution.

## Funding

N/A

## CRediT authorship contribution statement

MR and AH designed the paper. HO and HS collected the data, HO, and BIK wrote the first draft of the manuscript. FZ did the histopathology study. HS and AH participated in the article design and critically reviewed the manuscript. BIK, HS, AH and MR critically reviewed the manuscript. All authors approved the final version of the manuscript.

## Guarantor

Hamza Ouzzaouit.

## Declaration of competing interest

N/A
